# Vertical asymmetry of lamina cribrosa tilt angles using wide bandwidth, femtosecond mode-locked laser OCT; effect of myopia and glaucoma

**DOI:** 10.1007/s00417-016-3524-6

**Published:** 2016-10-28

**Authors:** Takuhei Shoji, Hiroto Kuroda, Masayuki Suzuki, Hisashi Ibuki, Makoto Araie, Shin Yoneya

**Affiliations:** 1 Department of Ophthalmology, Saitama Medical University, 38 Morohongo Moroyama-machi, Iruma, Saitama 350-0495 Japan; 2 Advanced Laser Medical Center, Department of Ophthalmology, Saitama Medical University, Iruma, Saitama Japan; 3 Department of Ophthalmology, Kanto Central Hospital, Tokyo, Japan

**Keywords:** Mode-locked laser, Optical coherence tomography, Lamina cribrosa, Myopia, Glaucoma

## Abstract

**Purpose:**

Morphological features of the lamina cribrosa (LC) and optic disc may be important in the pathogenesis of glaucoma and myopic neuropathy. We therefore performed a cross-sectional study of patients with glaucoma and myopic neuropathy to evaluate vertical asymmetry of LC tilt angles (LCTAs) from Bruch’s membrane opening (BMO).

**Material and methods:**

Forty-six control eyes and 35 primary open-angle glaucoma (POAG) eyes were included. A raster scanning protocol with 300 single B-scans (without averaging) were obtained using optical coherence tomography with a wide-bandwidth, femtosecond mode-locked (ML) laser. Superior temporal to inferior nasal (ST) direction and inferior temporal to superior nasal (IT) direction (±45° rotation with a horizontal line) lines were drawn, and the angle between the inner edge of the BMO plane and the best fitting line for the anterior LC plane was measured as the LCTA. The generalized estimating equation was used to analyze the eye-derived data.

**Results:**

Although no significant differences in either ST-LTCAs or IT-LTCAs were observed between the glaucoma group and non-glaucoma group, the IT-LCTAs were found to be significantly greater than the ST-LCTA in both the glaucoma and non-glaucoma groups (*P* < 0.001). After adjustment for other potential confounding factors by multivariate analysis, greater refractive errors were significantly correlated with IT-LCTAs.

**Conclusions:**

Vertical asymmetry of the LC tilting from the BMO plane exists in both normal and POAG eyes, and correlates with the degree of myopia.

## Introduction

Myopia is an independent risk factor for glaucoma, and is frequently associated with atypical retinal nerve fiber layer (RNFL) defects and nerve vulnerability [1, 2]. Furthermore, distortion of the lamina cribrosa (LC) is considered to be a major factor contributing to nerve vulnerability in myopia. Myopic eyes, particularly high myopic eyes, have stretching stress at the LC and at the edge of disc and sclera, which can cause susceptibility to nerve fiber damage [3, 4]. LC has also long been considered the primary site of axonal injury in glaucoma. Deformation and displacement of the LC has been increasingly implicated as the primary pathophysiological mechanism underlying glaucomatous optic neuropathy [5].

Optical coherence tomography (OCT) is a non-invasive optical imaging modality that allows real-time structural imaging of the fundus. Spectral-domain (SD) detection technology, combined with improvements in light sources incorporating a wider range of wavelengths (approximately 50 nm), has facilitated the development of currently available SD-OCT devices with three-dimensional (3D) imaging capability and high resolution (5–7 μm). Advancements in OCT have enabled detailed examinations of the deep optic nerve head (ONH), including the LC. In the clinical evaluations of ONH, the termination of Bruch’s membrane is considered the most consistent site from which to quantify the neuroretinal rim, and Bruch’s membrane opening (BMO) as detected by SD-OCT is considered a stable landmark and the most consistent outer border of the neuroretinal rim [6, 7]. Although posterior displacement between BMO and the anterior surface of the LC has been well studied in glaucoma [8, 9], there have been relatively few in-vivo studies demonstrating other anatomical parameters associated with BMO and LC in human eyes.

We identified that Bruch’s membrane terminates with single B-scanning (without averaging) using a high-resolution OCT system and a light source spectral bandwidth of 200 nm with relative ease [10]. High-resolution image acquisition enabled faster clearance of single-scanned images, resulting in substantially faster 3D image construction than that obtained with the standard commercial SD-OCT instruments that are currently available [11]. Using this system, we previously reported a novel indicator of LC tilt angles (LCTAs), which correlated with both glaucoma and refractive errors [12].

The purpose of the present study was two-fold, i.e., to investigate the vertical asymmetry of LCTAs, and to evaluate any potential correlations with disorders such as glaucoma and with refractive errors.

## Material and methods

The Ethics Committee of Saitama Medical University approved this cross-sectional comparative study, which was conducted in accordance with the tenets of the Declaration of Helsinki. Patients were included if they were at least 20 years old, fulfilled the eligibility requirements detailed below, and signed an informed consent form at a visit between April 2012 and July 2012.

### Inclusion criteria

To be eligible for this study, patients with glaucoma had to have been previously diagnosed with POAG involving characteristic glaucomatous ONH damage, such as localized or diffuse neuroretinal rim thinning associated with glaucomatous loss of the visual field, in accordance with the criteria of Anderson and Patella [13] using a Humphrey Field Analyzer (Carl Zeiss Meditec, Dublin, CA, USA) and the standard 30–2 program of the Swedish Interactive Threshold algorithm.

Control subjects were enrolled during the study period; they had to have intraocular pressures between 10 and 21 mmHg, no family history of glaucoma, a normal open anterior chamber angle, clinically normal appearance of the optic disc, and normal visual field results [13].

The subjects were divided into a highly myopic group (spherical equivalent, equal or worse than −5.0 D) and a non-highly myopic group (spherical equivalent, better than −5.0 D) on the basis of spherical equivalents.

### Exclusion criteria

Exclusion criteria were as follows: (1) visual acuity worse than 20/40, (2) poor reliability on visual field test result (>20 % fixation loss or >15 % false positive or false negative answers), (3) any other ophthalmic disease, including media opacity, diabetic retinopathy, neuro-ophthalmological disease, uveitis, ocular trauma, retinal, or choroidal disease, (4) other diseases capable of causing VF loss or optic nerve deterioration or a history of intraocular surgery or laser treatment, or (5) eyes with an extremely tilted or anomalous disc.

### Instruments

The OCT system was built by the Advanced Laser Medical Center (ALMC) at Saitama Medical University. The details of our SD-OCT system have been previously described elsewhere [11, 12, 14]. In brief, the OCT system used an ultra-broadband, Kerr lens, mode-locked (ML) Ti:sapphire laser, and a wide-band spectrometer. The spectral bandwidth of the light source was 200 nm full width at a central wavelength of 840 nm. A high speed CCD camera with 2048 × 300 pixels (Basler, Ahrensburg, Germany) was used as the detection system. The measurement speed was 50,000 depth-scans/s, and depth resolution was measured to be less than 2.0 μm into the tissue [12, 14]. The interferometer was attached to a semi-custom fundus scanning head system.

### Acquisition of in-vivo three-dimensional OCT images

A raster scanning protocol with 300 single B-scans with 300 A-scans (with 2048 pixels/A-Scan) covering a 3.0 × 3.0 mm square region centered on ONH was used for volumetric scans. Volumetric rendering of the 3D-OCT data set was performed, and longitudinal and en face cross-sections were constructed using an image processing software (Amira 5.4.3, Mercury Computer Systems Inc., Chelmsford, MA, USA). A fundus image was generated as an en face projection image from the 3D data set by integrating the magnitudes of the OCT signals at each lateral position along the axial direction. The total data acquisition time for a single 3D-OCT volumetric image was 3.0 s. OCT images were acquired after pupil dilation with tropicamide and phenylephrine hydrochloride (Mydrin P; Santen, Osaka, Japan). The optic disc was also imaged using a digital 30° fundus camera (Zeiss FF450, Carl Zeiss, Jena, Germany) immediately prior to OCT data acquisition.

### LCTA measurement

**Fig. 1:**
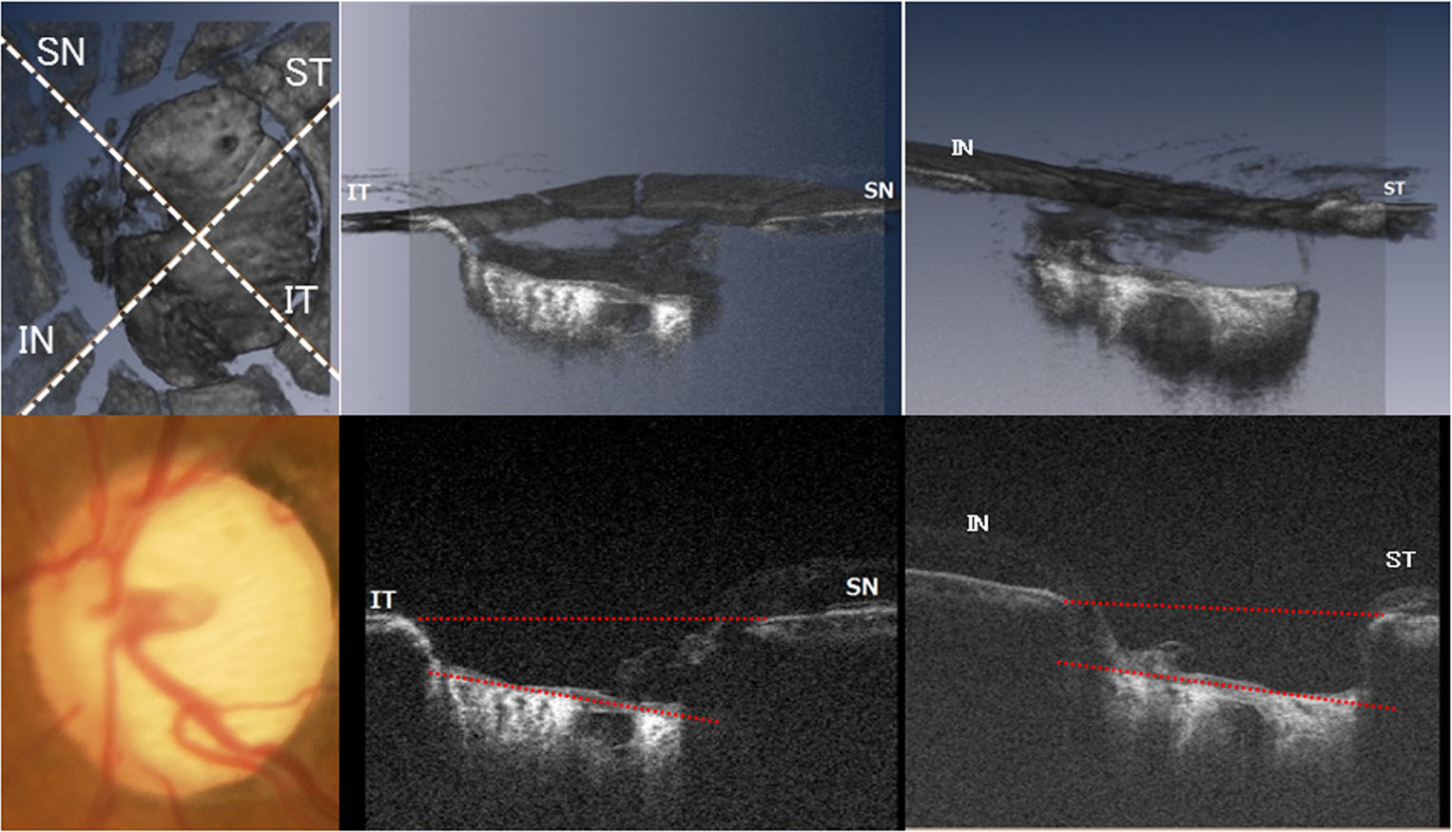
A schematic explanation of the methodology used for measuring the lamina cribrosa tilt angles (*LCTA*) against the Bruch’s membrane opening (*BMO*). *Top left*: lines were drawn in the superior temporal (*ST*) to inferior nasal (*IN*) and inferior temporal (*IT*) to superior nasal (*SN*) directions (±45° rotation with the horizontal line). *Bottom left*, *white dotted line*: fundus photography (top center and right) volume image sectioned at the level of IT to SN direction and ST to IN direction reconstructed from the 3D OCT dataset (*bottom center and right*) B-scans along IT to SN and ST to IN directions. A line (*red dotted line*) connecting the proximal tips of Bruch’s membrane on each side of the ON head on the cross-sectional SD-OCT image was drawn and set as the BMO reference plane. LCTA was defined as the angle measured between the inner edge of the BMO plane and the best-fitting line (*red dotted line*) for the anterior LC plane

The measurement of LCTAs was conducted as previously reported [12]. Briefly, following acquisition of an in-vivo 3D dataset, lines were drawn in a superior temporal (ST) to inferior nasal (IN) direction and inferior temporal (IT) to superior nasal (SN) direction (±45° rotation with the horizontal line). B-scan images of the ST-IN and the IT-SN directions were reviewed. Figure 1 shows a schematic explanation of the methodology used for measuring LCTA against BMO. We defined the ST-IN LCTAs (ST-LCTA) and IT-SN LCTAs (IT-LCTA) as follows. The center of the B-scan (white dotted line) was defined as the intermediate scan between the interruption where the Bruch’s membrane line started. A line (red dotted line) connecting the proximal tips of Bruch’s membrane on each side of the ON head on a cross sectional SD-OCT image was drawn and set as the BMO reference plane. LCTA was defined as the angle between the inner edge of the BMO plane, and the best-fitting line for the anterior LC plane measured as LCTA. The anterior surface of LC on B-scans was defined as the highly reflective plate structure underlying the optic disc cup. The definition of anterior surface of LC has been used in a previous ophthalmological study [15]. Angle measurements were performed with Amira software.

Measurements were repeated three times and averaged to provide final LCTA values. The intraobserver and interobserver reproducibility of LCTA measurements were evaluated in ten randomly selected eyes from five glaucomatous eyes and five normal eyes. Analysis was based on three independent series of re-evaluations made by two independent observers. The agreement of a single observer’s measurement and the mean of all three measurements conducted by the two observers were calculated with the intraclass correlation coefficient (ICC) using a 2-way mixed effect model.

### Statistical analyses

Data are expressed as means and the standard deviation (SD), with medians and interquartile ranges for continuous variables, and as frequencies with percentages for categorical variables. Baseline characteristics were summarized according to treatment sequences and compared using an unpaired *t*-test, the Wilcoxon rank sum test for continuous variables, or the chi-square test or Fisher’s Exact test for categorical variables, as appropriate. As a number of subjects had both eyes included in the study, the generalized estimating equation was used to analyze data derived from these eyes. This model was also used to evaluate the mean difference between the groups and has been used in a previous ophthalmological study [16].

To determine the effects of various factors on IT-LCTA and ST-LCTA, we performed univariate and multivariate regression analyses. The model was adjusted for candidate factors including age, sex, glaucoma, and refractive errors. Coefficients with 95 % confidence intervals (95 % CIs) were calculated. A *P*-value less than 0.05 indicated a statistically significant difference. All statistical analyses were performed using JMP version 10.1 software (SAS Institute, Inc., Cary, NC, USA) and SPSS software, version 22 (Japan IBM, Tokyo, Japan).

## Results

**Fig. 2:**
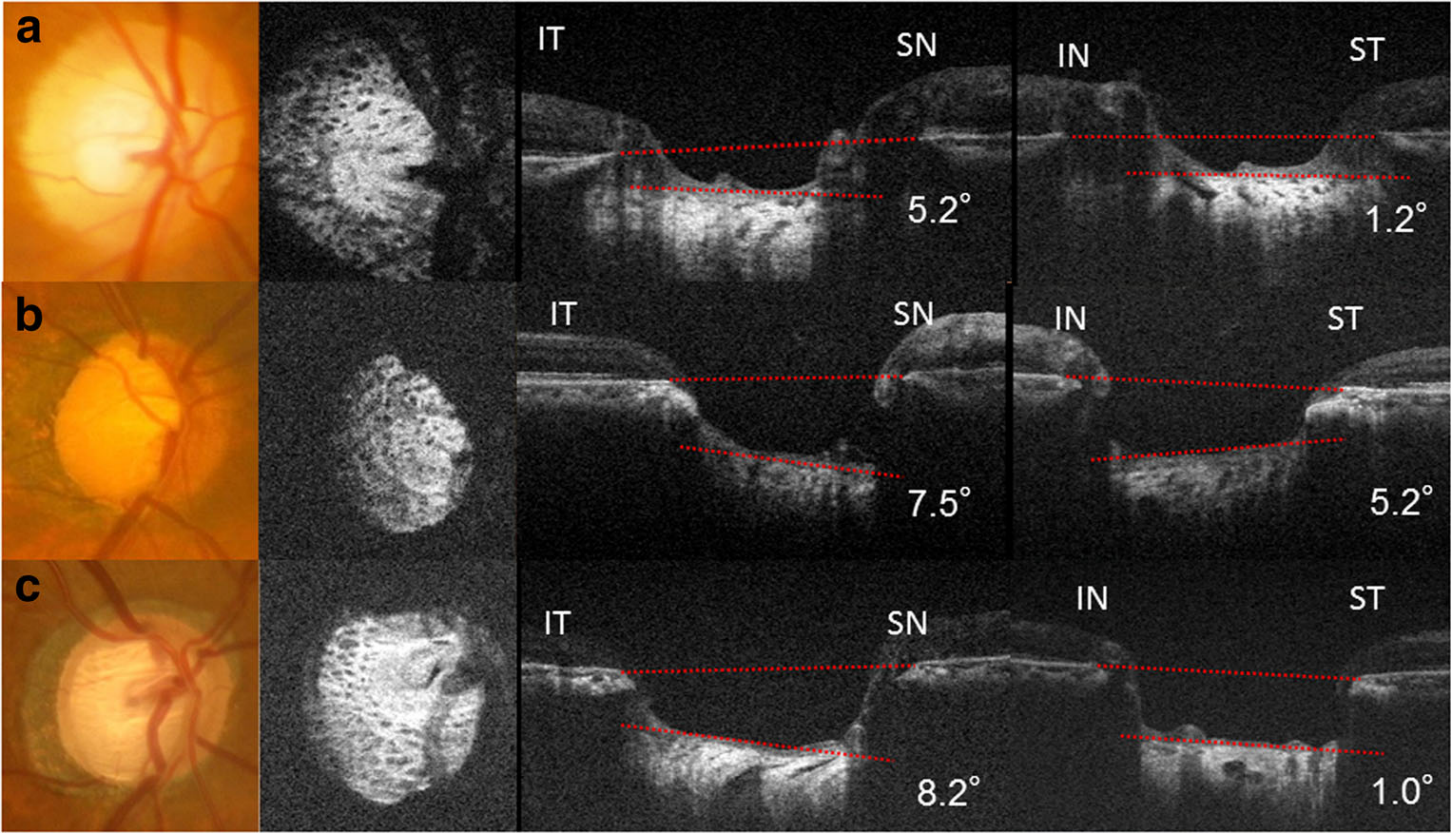
Fundus images and en face images from optical coherence tomography (*OCT*), and inferotemporal (*IT*) to superonasal (*SN*) direction and inferonasal (*IN*) to superotemporal (*ST*) direction B-scan images from optical coherence tomography (*OCT*). **a** (*top*) Images from the right eye of a 61-year-old male without glaucoma. The refractive error was +1.25D (*top right*). The LC tilting was mild in both the IT-SN direction (IT-LCTA) and the ST-IN direction (ST-LCTA). **b** (*middle left*) Images from the right eye of a 71-year-old male with glaucoma. The refractive errors were −6.00D and the visual field mean deviation was −15.22 decibels. Both IT-LCTA (*middle center*) and ST-LCTA (*middle right*) were evident. **c** (*bottom left*) Images from the right eye of a 73-year-old male with glaucoma. The refractive errors were −1.00D, and the visual field mean deviation was −15.52 decibels. IT-LCTA was greater than ST-LCTA. IT-SN (*bottom center*) and ST-IN (*bottom right*) views of the B-scan image

**Fig. 3:**
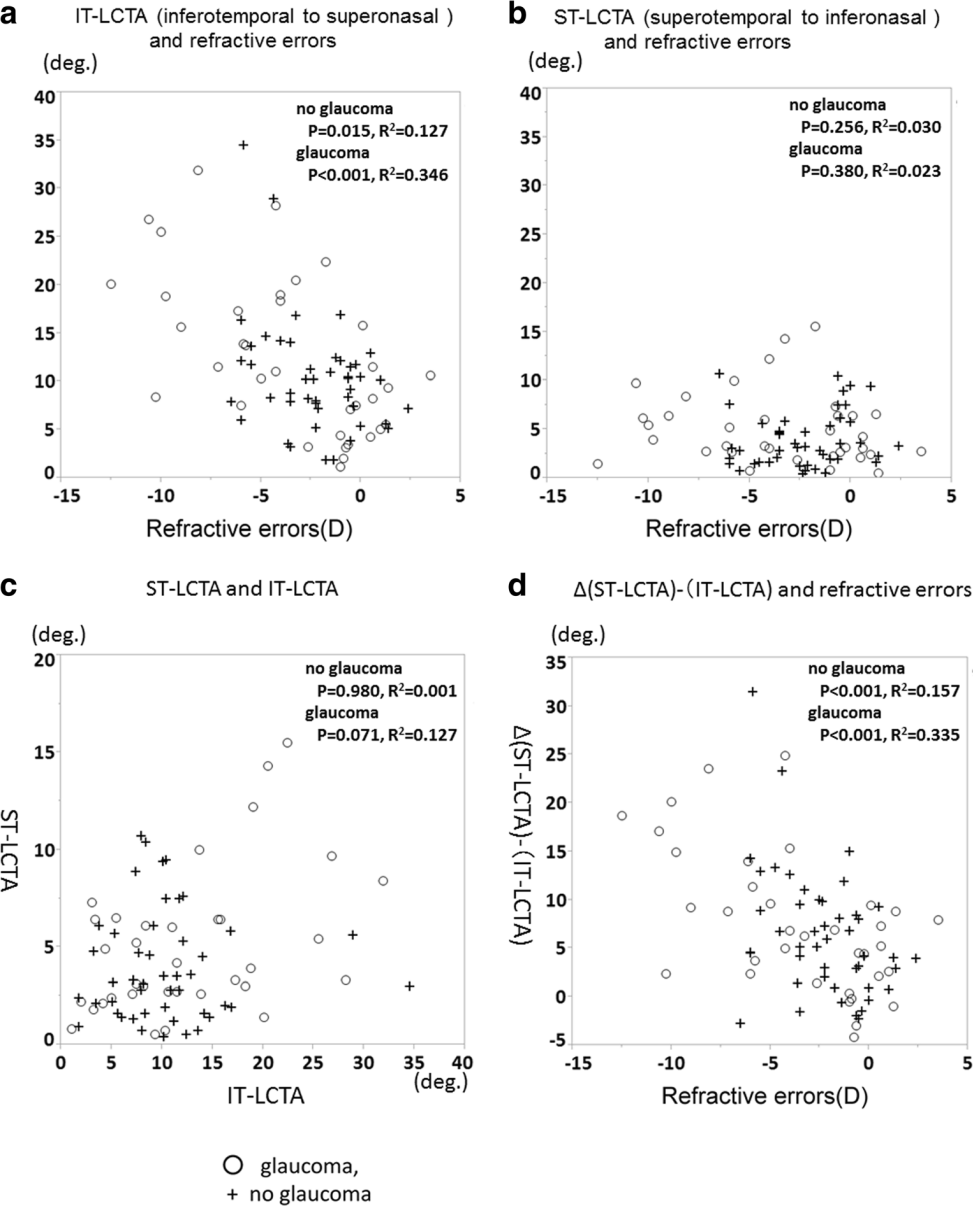
Scatterplot graphs of **a** IT-LCTA versus refractive errors and **b** ST-LCTA versus refractive errors. **c** Scatterplot graphs of IT-LCTA versus ST-LCTA. **d** Scatterplot graphs of the difference between ST-LCTA and IT-LCTA versus refractive error. *Circle*, with glaucoma; *cross*, without glaucoma

A total of 84 eyes from 48 participants were enrolled in the study. Of these eyes, three were excluded due to improper OCT images owing to media opacities and inappropriate fixation. Therefore, 81 eyes (from 46 participants), 46 normal eyes (25 subjects) and 35 primary open-angle glaucoma (POAG) eyes (21 patients), were included in the study. Table 1 summarizes the baseline characteristics of the study subjects. Mean LCTA measurements demonstrated excellent intra-observer (ICC = 0.930 for observer 1 and ICC = 0.969 for observer 2) and inter-observer (ICC = 0.983) agreement (all *P* < 0.001). Table 2 shows LCTAs from the BMO plane in both groups. The median (interquartile range) ST-LCTA and IT-LCTA were 3.2 (1.6–5.7) degrees and 10.1 (7.2–12.2) degrees respectively, in no glaucoma eyes, and 3.9 (2.6–6.4) degrees and 11.0 (5.5–18.8) degrees respectively, in glaucomatous eyes. Although no significant differences in either ST-LCTA or IT-LCTA were observed between the glaucoma group and non-glaucoma group, the IT-LCTAs were found to be significantly greater than the ST-LCTAs in both the glaucoma and non-glaucoma groups (*P* < 0.001). Moreover, although the difference in ST-LCTAs between group with high myopia and group without high myopia was not significant in both the glaucoma group and non-glaucoma group, IT-LCTAs in the group with high myopia were significantly greater than the group without high myopia in both the glaucoma group and non-glaucoma group. Table 3 shows the correlation between LCTAs and clinical and biometric parameters. Although there was no correlation between age, IOP, MD, or any LCTAs, there was a significant association between greater refractive errors with IT-LCTA and differences between ST-LCTA and IT-LCTA. Figure 2 shows representative cases. The top images are the hypertrophic eyes from a 61-year-old male patient without glaucoma. The middle images are from highly myopic eyes with glaucoma. The refractive errors were −6.00 D. The bottom images are from the moderately myopic eyes with severe glaucoma. The visual field mean deviation was −15.52 decibels. IT-LCTA was greater than ST-LCTA in these three images. Figure 3a and b show the scatter plots of refractive errors and IT-LCTA and ST-LCTA values. The IT-LCTA values were positively correlated with refractive errors in both the non-glaucoma group (*R* ^2^ = 0.127, *P* = 0.015) and the glaucoma group (*R* ^2^ = 0.346, *P* < 0.001), but no significant correlation was observed between the ST-LCTA and refractive errors. Figure 3c shows the scatter plots for ST-LCTA and IT-LCTA values. Figure 3d shows the scatter plot of significantly correlated differences between ST-LCTA and IT-LCTA values and refractive errors (*P* < 0.001). Table 4 describes the results of the multivariate analyses for factors potentially affecting ST-LCTA and IT-LCTA values. After adjusting for other potential confounding factors by multivariate analysis, greater refractive errors were significantly correlated with IT-LCTA (coefficient, −1.27 per diopter; *P* < 0.001) and the differences between IT-LCTA and ST-LCTA (coefficient, −1.14 per diopter; *P* < 0.001). The interactions between refractive errors and POAG did not show statistically significant correlations with IT-LCTA using multivariate analyses.

**Table 1:** Baseline characteristics of the group without glaucoma and the group with glaucoma

	No glaucoma group (*n* = 46)	Glaucoma group (*n* = 35)	*P* value
Male sex (*n*, %)	33 (71.7)	23 (63.9)	0.631
Age (years)	44.5 ± 16.9	63.1 ± 8.4	<0.001
Spherical equivalent error (D)	−2.2 ± 2.3	−3.7 ± 4.1	0.067
IOP (mmHg)	14.9 ± 2.3	17.2 ± 5.2	0.010
MD (dB) (median [25,75 percentile])	0.08 (−1.76, 0.73)	−11.2 (−5.0, −18.12)	<0.001

Plus–minus values are means ± SD

Baseline characteristics were compared with the unpaired t-test between the groups

Abbreviations: *D* diopters, *IOP* intraocular pressure, *MD* mean deviation, *dB* decibels

**Table 2:** Lamina cribrosa tilt angles in each group

	LC tilt angle from BMO plane (deg.) (median [25,75 percentile])				
	Superotemporal to inferonasal direction	*P* value*	Inferotemporal to superonasal direction	*P* value*	*P* value^†^
No glaucoma group					
Overall (*n* = 46)	3.2 (1.6, 5.7)		10.1 (7.2, 12.2)		<0.001
No high myopia (*n* = 39)	3.3 (1.6, 5.7)		9.2 (7.2, 11.7)		<0.001
High myopia (*n* = 7)	2.8 (1.4, 7.6)		12.1 (7.9, 16.3)		0.016
*P* value^‡^	0.965		0.030		
Glaucoma group					
Overall (*n* = 35)	3.9 (2.6, 6.4)	0.176	11.0 (5.5, 18.8)	0.300	<0.001
No high myopia (*n* = 22)	3.2 (2.4, 6.4)	0.428	7.9 (4.0, 16.4)	0.625	<0.001
High myopia (*n* = 13)	5.2 (2.7, 7.4)	0.428	15.6 (10.9, 22.8)	0.322	<0.001
*P* value^‡^	0.657		0.012		

Abbreviations: *LC* lamina cribrosa, *BMO* Bruch’s membrane opening, *deg* degrees

* P value for no glaucoma group vs glaucoma group

†* P* value for superotemporal to inferonasal direction vs inferotemporal to superonasal direction

‡* P* value for no high myopia group vs. high myopia group

**Table 3:** Association of potential clinical and biometric parameters with the lamina cribrosa tilt angle

	Lamina cribrosa tilt angle					
	Superotemporal to inferonasal direction		Inferotemporal to superonasal direction		Difference between IT-LCTA and ST-LCTA	
Variables	Coefficients	*P* value	Coefficients	*P* value	Coefficients	*P* value
Age (years)	0.02	0.419	−0.01	0.771	−0.03	0.496
IOP (mmHg)	−0.05	0.571	−0.05	0.809	0.00	0.983
MD (dB)	−0.12	0.073	−0.08	0.558	0.04	0.779
Refractive errors (SEQ)	−0.07	0.529	−1.12	<0.001	−1.05	<0.01

Abbreviations: *IOP* intraocular pressure, *MD* mean deviation, *dB* decibels, *SEQ* spherical equivalent, *D* diopter, *IT* inferotemporal to superonasal direction, *ST* superotemporal to inferonasal direction

**Table 4:** Association of potential clinical and biometric parameters with the lamina cribrosa tilt angle based on multivariate analyses

	Superotemporal to inferonasal direction		Inferotemporal to superonasal direction		Difference between IT-LCTA and ST-LCTA	
Variables	Coefficients	*P* value	Coefficients	*P* value	Coefficients	*P* value
	(95% CI)		(95% CI)		(95% CI)	
Age (per year)	0.02 (−0.09, 0.12)	0.755	0.11 (−0.16, 0.38)	0.422	0.10 (−0.16, 0.35)	0.459
Sex (reference: male)	−1.62 (−3.96, 0.71)	0.173	0.31 (−5.63, 6.24)	0.919	1.93 (−3.52, 7.38)	0.487
MD (dB)	−0.11 (−0.29, 0.07)	0.215	0.06 (−0.29, 0.33)	0.744	0.16 (−0.08, 0.40)	0.193
Refractive errors (SEQ) (per D)	−0.03 (−0.22, 0.17)	0.799	−1.27 (−1.90, −0.64)	<0.001	−1.14 (−1.77, −0.46)	<0.001

Abbreviations: *MD* mean deviation, *dB* decibels, *SEQ* spherical equivalent, *D* diopter, *IT* inferotemporal to superonasal direction, *ST* superotemporal to inferonasal direction

## Discussion

The principal findings of this report were that the inferotemporal LCTA (IT-LCTA) was significantly larger than the superotemporal LCTA (ST-LCTA), and greater IT-LCTA and differences between them were significantly correlated with greater refractive errors after adjustment for other potential confounding factors, including presence of POAG. These results indicate that morphological features of LC, namely vertical asymmetry LC tilting in the IT-SN direction with reference to BMO, were correlated with refractive errors. In contrast, no correlation was found between LC tilting in the ST-IN direction, and either presence of POAG or refractive errors.

The measurements in the present study were facilitated by SD-OCT with a wide-broadband light source that was able to depict the surface of the LC and BMO with a single scan with less than 2 μm axial resolution. In a report by Park et al. [17], it took 10–20 min to construct a 3D image, which may preclude the use of commercial SD-OCT instruments for screening purposes. The theoretical advantage of a ML laser compared with superluminescent diodes (SLD) as an OCT light source [14] made the total data acquisition time for a single 3D-OCT (volumetric) image with shorter than the SD-OCT instrument using SLD. Although commercial swept-source (SS)-OCT instruments with a central wavelength of 1050 nm are able to provide deep penetration to the LC, the axial resolution is inferior to commercial SD-OCT instruments using SLD (see manufacturer’s homepage at http://www.topcon-medical.eu/eu/products/177-dri-oct-1-atlantis-swept-source-oct.html#specifications) due to the bandwidth and central wavelength of the light source.

Myopia has been reported to be associated with optic disc tilting [18]. Kim et al. suggested tilted optic discs in myopic eyes may be an acquired feature resulting from scleral stretching in the parapapillary region associated with myopic shift [19]. In some myopic eyes, particularly highly myopic eyes, a number of abnormalities may be observed around the ONH, including peripapillary conus, intrachoroidal cavity, optic disc pit, and focal LC defects [20–22]. Although the pathogenesis of peripapillary intrachoroidal cavity has yet to be fully elucidated, the intrachoroidal cavity commonly involves the area inferior to the optic disc [23]. Our observations also have implications for the current understanding of the pathogenesis of peripapillary intrachoroidal cavity in highly myopic eyes, as intrachoroidal cavity has previously been considered a temporal ONH change due to axial elongation.

Focal LC defects were defined as defects of continuous LC hyper-reflectivity. Although the locations of these focal defects corresponded with those of RNFL defects and disc hemorrhages [21], the mechanism of focal LC defect formation has yet to be elucidated. However, the majority of focal LC defects occurred in the inferior or inferotemporal periphery of LC [24], and the frequency of LC defects was much higher in eyes with high myopia than in those without. Recently, Kimura et al. reported that the formation of LC defects may be associated with temporal elongation of the optic disc; he demonstrated that the presence of LC defects was significantly associated with disc deformation, such as vertical “disc” tilt angle, by multivariate analysis [25]. Our results corroborate these previous findings, demonstrating that structural changes in the inferotemporal region are associated with myopia. In this study, greater refractive errors were correlated with greater IT-LCTA, possibly due to stretching or deformation, involving lamina tilt in the axial direction in addition to disc tilt. We previously reported that LCTA were correlated with glaucoma in the vertical direction and refractive errors in the horizontal direction [12]. We hypothesize that LC tilting from the BMO plane, particularly in the inferotemporal direction due to refractive errors, might be associated with deformation around the disc and LC such as peripapillary ICC or focal LC defects. Further, the inferotemporal sector of the neuroretinal rim is the most significant sector in patients with glaucoma. Nevertheless, the detailed mechanisms underlying these abnormalities remain unclear, with these theories currently remaining hypothetical. Further studies are required to fully evaluate any correlation between lamina tilt and disc deformation.

We used BMO as a reference plane to measure LCTA, but not the photographed disc margin, because photographically determined clinical disc margins reportedly represent complex anatomical structures that differ between individual eyes [6, 26, 27]. On the other hand, BMO detected by SD-OCT is considered to be the most consistent outer border of the neuroretinal rim and a stable landmark [6, 7]. Three-dimensional imaging allowed measurement of LCTA from the BMO plane along the ST to inferonasal and along the inferotemporal to superonasal lines through the optic disc center, determined by using the edges of BMO on the 3D dataset as references. However, BMO, in addition to scleral openings, might also be tilted in myopic eyes. It remains to be determined whether BMO tilting and LC tilting in myopia are dependent or independent phenomena. Our findings, taken together with previous findings, suggest that myopia results in LC deformation against BMO in addition to altering disc configuration on ONH.

Several limitations of this study warrant discussion. First, this was a pilot study involving a small number of subjects and had a cross-sectional design. The effect of progression of glaucomatous neuropathy on the LCTA might be underestimated because of the small number of cases and undetected progression of glaucoma. Refractive errors and age were not matched between the no-glaucoma group and the glaucoma group. To control the effects of age differences, we adjusted for potential factors, including age, mean deviation, and refractive errors using multivariate analysis. Further investigation using a greater number of subjects, a longitudinal design, and other potential factors (e.g., central corneal thickness) will be required to confirm our findings. However, the effect of refractive errors was statistically significant and unlikely to be overestimated due to sample size. Second, we measured LC tilting within the optic disc cupping as the reflectivity of LC beneath the neuroretinal rim was weaker than that within the cupping. To exclude the possibility of incorrect measurements, we chose not to use the LC surface line beneath the rim. Visualization of the LC surface beneath the rim, if possible, would enable more accurate determination of LCTA. Third, we were unable to correct the magnification effects on lateral measurement of fundus structures through OCT imaging, which could affect the results of angle measurement. According to Littman [28], uncorrected lateral measurement is decreased with increasing axial length. Therefore, the possibility that magnification effects were, at least in part, responsible for the correlation between LCTA and refractive errors cannot be excluded. We estimated the effects of magnification error on angle measurement and measurement error to be less than 15 % in the eyes of our current subjects. Thus, this effect would not have had a critical effect on our results as refractive errors were associated only with IT-LCTA using multivariate analysis, regardless of the identical magnification effects on IT-LCTA or ST-LCTA. However, the effects of axial length and refraction on morphological parameters should be investigated in further studies. In addition, the angles measured in our study using OCT should be viewed with caution, as they were calculated by imaging software and were not actual measured distances over the curvature of the eye. Nevertheless, our study is the first to report measurements obtained by OCT in eyes with myopia, and we believe that these measurements and associations will enhance our understanding of myopia and LC.

In conclusion, we obtained in-vivo 3D images of BMO and LC planes and measured inferior-temporal and superior-temporal tilt angles of LC plane from the BMO plane. Inferior-temporal LCTAs, and the difference between superior and inferior LCTAs, were found to be correlated with refractive errors. These results suggest that LC tilting against BMO was vertically asymmetrical, and the inferior temporal direction was more strongly correlated with refractive errors. These distortions may provide an additional explanation for the various disorders affecting the disc due to myopia; however, further studies are required to clarify the internal optic disc structural changes associated with this visual defect.
